# Population perspectives and demographic methods to strengthen CRVS systems: introduction

**DOI:** 10.1186/s41118-022-00156-8

**Published:** 2022-02-23

**Authors:** Romesh Silva

**Affiliations:** 1 Scientific Panel on Population Perspectives and Demographic Methods to Strengthen CRVS Systems, International Union of the Scientific Study of Population, Paris, France; 2 Population and Development Branch, Technical Division, United Nations Population Fund, New York, USA

**Keywords:** Civil registration, Birth registration, Marriage registration, Death registration, Vital statistics, Legal identity, Mortality, Fertility, Nuptiality, Population register

## Abstract

Civil registration and vital statistics (CRVS) systems and legal identity systems have become increasingly recognized as catalytic both for inclusive development and for monitoring population dynamics spanning the entire life course. Population scientists have a long history of contributing to the strengthening of CRVS and legal identity systems and of using vital registration data to understand population and development dynamics. This paper provides an overview of the *Genus* thematic series on CRVS systems. The series spans 11 research articles that document new insights on the registration of births, marriages, separations/divorces, deaths and legal residency. This introductory article to the series reviews the importance of population perspectives and demographic methods in strengthening CRVS systems and improving our understanding of population dynamics across the lifecourse. The paper highlights the major contributions from this thematic series and discusses emerging challenges and future research directions on CRVS systems for the population science community.

## The growing importance of civil registration and vital statistics systems in population studies and development

Civil registration is the continuous, permanent, compulsory, and universal recording of the occurrence and characteristics of vital events pertaining to the population, as provided through decree or regulation in accordance with the legal requirement in each country (United Nations, 2014). Civil registration and vital statistics (CRVS) systems are critical for protecting basic human rights; in facilitating inclusion in social, economic, and political life; and for understanding population and health dynamics (United Nations General Assembly, 2015). Yet, across the world, approximately 25% of births of children under 5 years are unregistered (UNICEF, 2019). Estimates of global unregistered deaths range from 40 to 60% and more than two-thirds of low-income countries have not yet established a standardized system of cause of death reporting (Mikkelsen et al., 2015; United Nations, 2021). Currently, there are no comprehensive estimates of marriage and divorce registration completeness. This situation has been described as a “scandal of invisibility” that fuels poverty and inequality and also hampers evidence-based policies and programs (Setel et al., 2007). Solving the pervasive problem of underregistration of vital events is central for poverty reduction, as children and adults who lack proof of legal identity are often denied access to health care, education, housing, nutrition, and other support through social protection programs (Szreter & Breckenridge, 2012).

The severe acute respiratory syndrome coronavirus 2 (SARS-CoV-2, or COVID-19) pandemic has further underscored both the importance of high-quality civil registration data and the power of demographic methods in understanding population and demographic change (Egidi & Manfredi, 2021). Timely and accurate death registration data have progressively informed our understanding of the mortality effects and consequences of the pandemic as it evolves (Kiang et al., 2020). Yet, the pandemic has also highlighted weaknesses in many CRVS systems around the world along with the need for systems-level improvements and rapid innovation (AbouZahr et al., 2021).

The perspectives of population analysis and the tools of demography have long been employed in the area of CRVS systems. Graunt pioneered the production, dissemination and use of routine mortality statistics, in the form of bills of mortality in seventeenth century London. This, in turn, led to a revolution in evidence-based public health (Morabia, 2013) and spurred innovation by William Farr and the General Registrar’s Office in England (Lilienfeld, 2007). Historical demographers and population historians have used early population-based registers to understand the historical trends in fertility, nuptiality, mortality, migration and intra-household dynamics (Alter et al., 2010; Bengtsson et al., 2004). Further, economic historians have demonstrated that identity registration and comprehensive social security systems are key facilitators of labor mobility and human capital formation (Szreter, 2007). More recently, proof of legal identity from birth through to death has been recognized as a key protection measure and enabler of opportunity in situations of forced displacement and of safe, orderly, and regular migration (Corneloup & Verhellen, 2020; Manby, 2016). Hence, there is a long history of demographic methods strengthening vital event registration systems and population analysis of civil registration data shaping public policy and public health action.

**Fig. 1:**
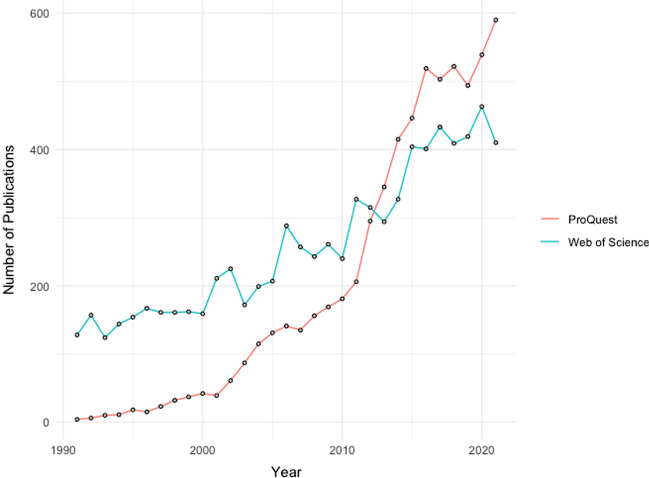
Peer-reviewed publications on civil registration and vital statistics in ProQuest and Web of Science databases, 1991–2021. Search criteria used was “civil registration” or “vital statistics”, based on systematic search criteria used in Abouzahr et al. (2015)

Efforts to strengthen civil registration systems and harness vital statistics to guide public policy have been accelerating over the past few decades. Political commitments to universal civil registration and representative vital statistics have been reaffirmed across a number of regions (United Nations Economic & Social Commission for Asia & the Pacific, 2021; United Nations Economic Commission for Africa, 2019b). International financing in support of CRVS system-strengthening as a supplement to domestic resources has grown in recent decades, and the estimated financing needed for CRVS systems strengthening between 2015 and 2024 was US$3.8 billion (World Bank Group & World Health Organization, 2014). Further, there has been a growing body of research on the evaluation of CRVS systems; their integration with other data systems such as censuses, population registers, surveys, and geospatial data systems, and the analysis of civil registration data and production of vital statistics. This can be seen in the growth in peer-reviewed articles on civil registration and vital statistics indexed in the ProQuest and Web of Science academic databases over the last two decades, as shown in Fig. 1.

A major emerging challenge in the field is ensuring that CRVS systems are effective, equitable, and sustainable. Bhatia et al. (2017) found major disparities between birth registration completeness and birth certificate possession in 41 countries. This disparity between registration and certification shows that even when vital events are officially registered, many people lack the associated certificate or proof of legal identity credential that is needed to access educational, health and social protection entitlements. Between 1999 and 2016, birth registration for children under 5 years improved in 53 out of 67 countries with available Demographic and Health Survey (DHS) and Multiple Indicator Cluster Survey data, yet subnational wealth inequities in birth registration declined in only 10 countries during this same period (Bhatiya et al., 2019). This highlights the uneven progress in ensuring that everybody counts both within and between countries. Ultimately, such invisibility, born out of system failure, impedes individual human rights and dampens overall social and economic progress.

## The thematic series “population perspectives and demographic methods to strengthen CRVS systems”

This thematic series was developed through the IUSSP Scientific Panel on Population Perspectives and Demographic Methods to Strengthen CRVS Systems. Many of the papers in this *Genus* thematic series were presented at research workshops and seminars in 2020–2021 hosted by the IUSSP Scientific Panel on CRVS Systems. Others were received as part of an open call for papers by *Genus*. The papers showcase research across several low- and middle-income countries and two high-income settings, and are drawn primarily from scholarship by IUSSP CRVS Fellows, members of the IUSSP Scientific Panel on CRVS Systems and their collaborators with the support of Global Affairs Canada, the IDRC Centre of Excellence on CRVS Systems, and the IUSSP Secretariat.

This thematic series showcases demographic methods and population perspectives that are becoming critical for the progressive strengthening and sustainable maintenance of robust civil registration and vital statistics systems. The 10 papers in this series span diverse areas of the globe including the Asia Pacific, sub Saharan Africa, Latin America, and Western Europe. They, in turn, present a series of subnational studies in Bangladesh, India, Italy, Malaysia, Senegal, nationally representative studies in Italy and South Africa, and a multi-country study on death registration and excess mortality in 6 Latin American countries.

## Life-course approach and population perspective

**Fig. 2:**
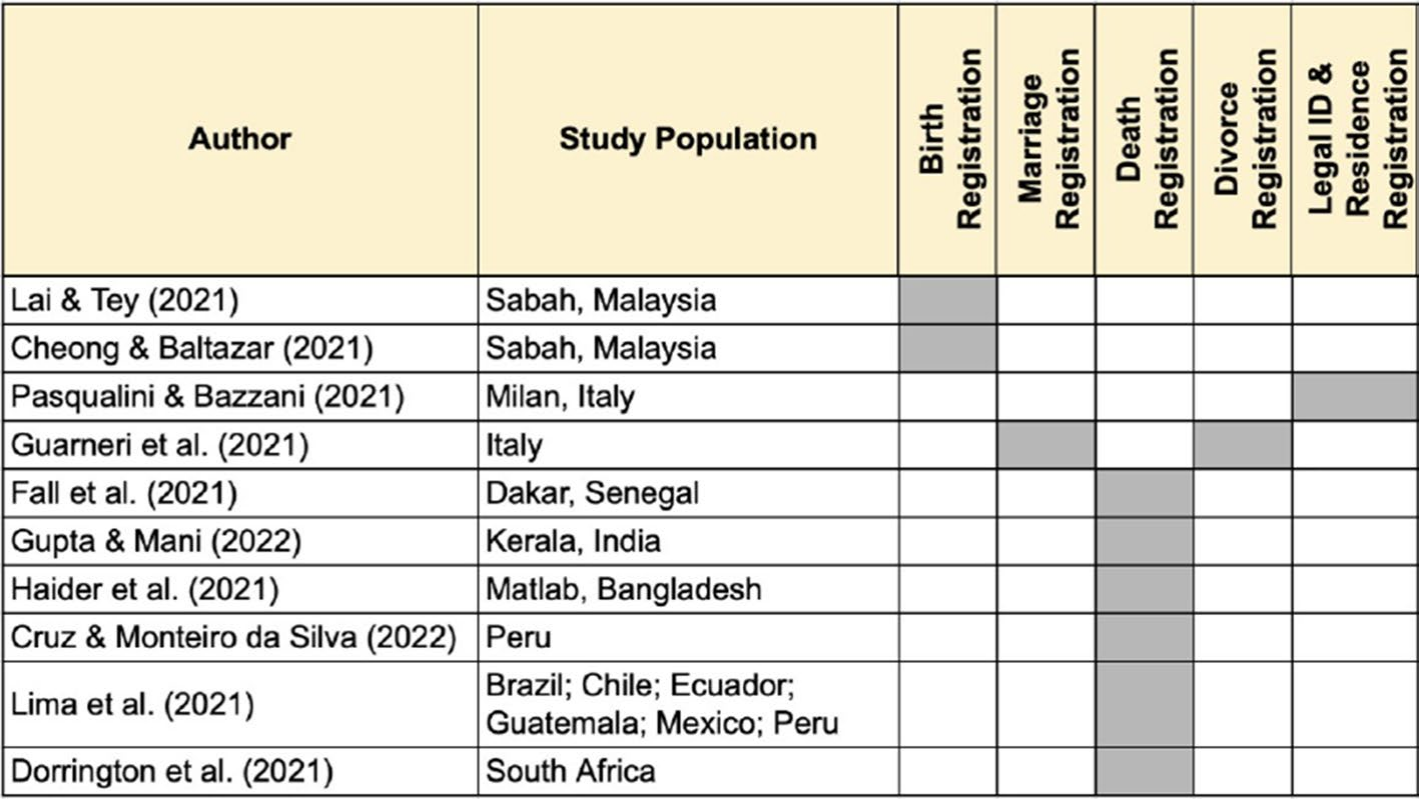
Engagement of *Genus* thematic series papers with different components of civil registration and vital statistics systems and legal identity systems

The series takes a life-course approach as the topics engaged span birth, marriage, divorce and death registration as well as residence registration and identity management. Read together, the thematic series papers highlight the enabling role that timely and accurate civil registration plays in promoting and protecting basic rights, supporting effective governance systems and administrative processes, and generating data for vital statistics analysis. Figure 2 notes the engagement by the thematic series papers across the full life-course and components of CRVS and legal identity systems.

Engagement of a full life-course approach makes sense both in terms of human rights and inclusive development strategies and also in terms of official statistics and public accountability. Civil registration and legal identity facilitate access to basic rights such as health care, primary and secondary education, and social support.

The underregistration of women’s vital events hinders gender equality and adds barriers to social and economic opportunity. Universal marriage and divorce registration, which are too often neglected, also facilitate access to rights irrespective of sex or gender orientation. A marriage certificate provides legal proof of marriage, which women can use to secure property and to collect an inheritance when their spouse dies. Similarly, divorce registration allows both parties to remarry after a divorce, and provides a legal basis for distributing parental responsibilities at the end of a marriage. Death registration data are a critical source of mortality statistics. The underregistration of female deaths relative to male deaths hinders the accuracy of evidence-based health programs for women and girls. This strategic importance of strong CRVS systems is well recognized in the United Nations’ 2030 Sustainable Development Agenda, specifically in Sustainable Development Goal target 16.9 which affirms the importance of universal legal identity, and SDG target 17.19 which emphasizes the value of CRVS systems alongside population and housing censuses (United Nations General Assembly, 2015).

This *Genus* thematic series highlights the gender dimensions of CRVS systems—through the study of sex-specific differentials in completeness of registration of vital events (Haider et al., 2021), and through the analysis of gender aspects that shape the laws, social norms, and bureaucratic processes related to civil registration systems (Cheong & Balthazar, 2021; Fall et al., 2021).

**Fig. 3:**
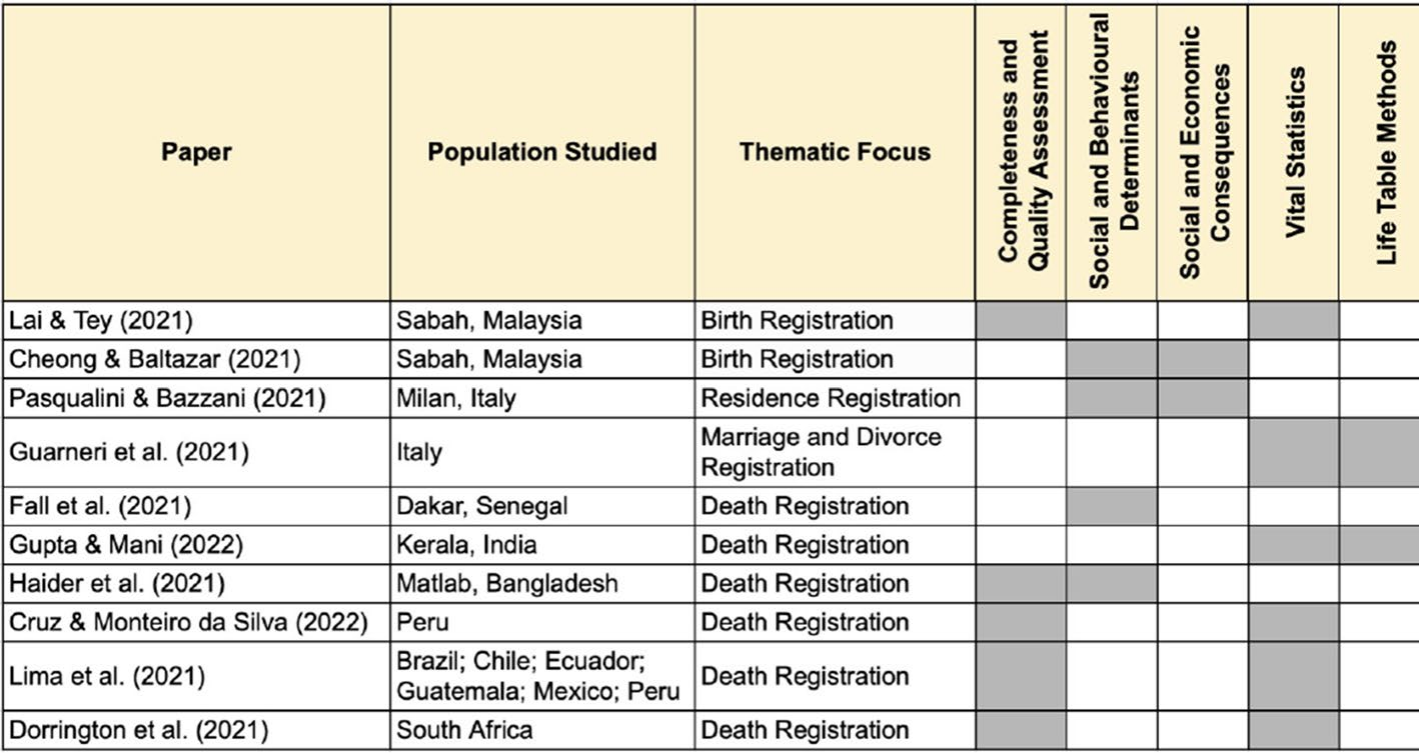
Methodological approaches employed by papers in the *Genus* thematic series on civil registration and vital statistics systems

The papers in the series employ a rich array of methodological approaches to both assess civil registration systems and utilize the underlying data to understand the levels, trends and nature of population change. Figure 3 describes the range of analytical approaches used in the thematic series, which span qualitative, mixed methods, and formal statistics and demographic methods. This range of technical approaches exemplifies the complex challenges of understanding and using civil registration data as well as the multifaceted nature of CRVS systems.

## Review of papers in the *Genus* thematic series

### Birth registration in Sabah, Malaysia

The first two papers in the *Genus* thematic series on CRVS systems investigate the extent, determinants and consequences of birth underregistration in Sabah, Malaysia. The first paper, by Lai and Tey (2021), uses birth registration data and the most recent census data to show that one of the largest geographic areas of Malaysia, which is more sparsely populated, and has a higher proportion of foreign labor migrants, has substantial birth registration incompleteness. The authors use a new indirect demographic method that requires only high-quality census data disaggregated by age and sex. The authors use the age- and sex-structure of the population census data to derive a gold standard measure for age-specific and total fertility rates for districts/subdistricts in Sabah, Malaysia. This study highlights the importance of interrogating seemingly “complete” civil registration data, such as those of Malaysia, and carrying out specific assessments for vulnerable subpopulations in small subnational areas to identify gaps and deficiencies in the civil registration system.

The findings by Lai and Tey (2021) provide important insights for national and local authorities in Malaysia, civil society, and local community leaders seeking to strengthen birth registration in Sabah, Malaysia. Their approach also provides opportunities for demographers to replicate inequality assessments of birth registration access in subnational areas using widely available aggregate data from population and housing censuses and civil registries. Finally, their study provides practical guidance on how the methods and tools of demography can be applied to identify gaps in birth registrations for small, localized subpopulations.

In the second paper of the thematic series, Cheong and Baltazar (2021) investigate the impediments to birth registration and maternal health access in Sabah among irregular migrants and stateless persons. The authors highlight the importance of unpacking the political economy of civil registration when seeking to understand barriers and factors impeding universal birth registration. They employ a novel qualitative approach, the document inventory method that synthesizes semi-structured interview data and documentary evidence about a person’s identity. In particular, the authors note how individual decisions by foreign labor migrants about engagement with state-based information systems can shape their access to proof of legal identity and access to maternal and newborn child health services. Their work highlights the notable barriers and vulnerabilities faced by undocumented labor migrants, stateless persons, and ethnic minorities in attaining proof of legal identity and participating fully in social, economic, and political life (Harbitz & Tamargo, 2009; Manby, 2020).

Cheong and Baltazar (2021) demonstrate the value of qualitative methods, such as the document inventory method, in assessing social and behavioral impediments to legal identity for foreign labor migrants in a subregion of Malaysia, a country that has reported universal birth registration for some time. The authors unpack the complex relationships between underregistration of births and impediments to maternal and child health using the “three delays model” (Thaddeus & Maine, 1994). Their findings highlight the connections between proof of legal identity, universal health coverage (SDG 3), gender equality (SDG 5), and just, peaceful and inclusive societies (SDG 16), in general. They also identify political, legal, and institutional determinants of underregistration among stateless persons, irregular migrants, and their descendants.

Cheong and Baltazar (2021) offer a new approach to evaluating the inclusiveness of legal identity systems for vulnerable subpopulations. Their work expands the growing tool kit of social scientific methods that can be used to unpack the determinants and consequences of vital event underregistration—ranging from multivariate regression analyses of household survey data (Nomura et al., 2018; Wakibi & Ngure, 2021), to semi-structured interviews of vulnerable subpopulations and key informants (Jewkes & Wood, 1998), to behavioral analysis informed by a socioecological model of vital event registration determinants (United Nations, 2019a, 2019b). The document inventory method, coupled with the three-delays framework to understand political and legal barriers to birth registration, also offers important small-scale research tools to investigate barriers to registration of vulnerable subpopulations as a complement to well-established direct and indirect demographic assessment methods that draw on census, survey and administrative data sources (Hill, 2017).

## Proof of legal identity, residence registration and access to social protection

Pasqualini and Bazzani (2021) investigate the important issues of societal inclusion and social protection of homeless persons in Milan, Italy, by studying the challenges and opportunities of residence registration for homeless persons. Their qualitative study highlights the importance of evaluating access to proof of legal identity in high-income settings—specifically as a means to understanding social exclusion and addressing homelessness. This paper focuses on the invisibility and exclusion inherent to homelessness and highlights the important role that residence registration and proof of legal identity have in facilitating access to basic social services for the homeless.

Pasqualini and Bazzani (2021), similar to the study by Cheong and Baltazar (2021) of foreign labor migrants in Sabah, Malaysia, find that foreign nationals in Milan face additional challenges in accessing residence registration. They specifically highlight that when an individual is not recorded in the residence population registry, access to basic social and health services is severely reduced. In fact, without residence registration, it is difficult for a homeless person to obtain proof of legal identity in Italy. This study identifies three main barriers to residence registration in Milan: (a) the lack of awareness among homeless persons and homeless service providers of the importance of residence registration; (b) fear and mistrust, particularly among foreign-born homeless persons, of service providers and government registration practices; and (c) social stigma and social exclusion, which impede trust in institutions and visibility that are inherent in the process of residence registration. Although their study is limited in its generalizability, this paper notes the role of legal identity and residence registration in protecting the rights of the homeless and facilitating access to basic social and health services. This research is a notable example of the importance of inclusive legal identity systems in supporting vulnerable subpopulations via social protection programs (Harbitz, 2013; Tamargo et al., 2020). The paper also highlights the importance of systematically evaluating the accessibility of legal identity systems for vulnerable subpopulations, even in high-income countries where aggregate national statistics suggest that the civil registration and legal identity system is complete.

## Adapting classical demographic methods to the study of marriage registration and marital separation

In the fourth paper in the thematic series, Guarneri et al. (2021) use marriage registration and administrative records on separation and divorce to quantify the magnitude, frequency and nature of marriage disruption dynamics in Italy. Studying the factors that affect marital disruption is challenging due to the complexity of the juridical process of marital separation and its variation over time, the relative rarity of marital disruption, and the challenge of constructing complete marital histories. The authors find that separation risk across marriage cohorts has grown between 1975 and 2015 and that marital disruption risk varies by selected characteristics of partners and of the marital union. They find that marital disruption risk varies depending on whether the marriage is a civil or religious marriage, property regime, and geographic area and the couples’ citizenship at birth.

The study by Guarneri et al. (2021) is innovative in that it adapts classical demographic life-table methods used to study mortality dynamics to illuminate an understudied area of vital statistics—marital separation and divorce. This study demonstrates how aggregate marriage registration data can be linked to administrative records on marital disruption to produce detailed vital statistics analyses of marital dissolution dynamics. The authors outline future prospects of extending this work further via microdata linkage of marriage registration data, administrative records on marital separation, and official statistical registers. Such data integration work will provide a foundation for more detailed understandings of marital duration and dissolution dynamics by employment characteristics, income levels, and educational attainment.

## Applications of demographic and analytical methods to the study of death registration data and mortality estimation

In the fifth paper of the series, Fall et al. (2021) examined differences in death registration completeness in different small-area neighborhoods of Dakar, Senegal. The authors used multiple data sources; the most recent population and housing census, a survey of relatives of recently deceased persons, and geospatial data. They find that underregistration of deaths is mostly associated with household-level poverty, low educational attainment, and higher population density per civil registration center. In contrast to earlier assessments that documented notable subnational sex differentials in death registration completeness in Ecuador, Morocco, and Bangladesh (Haider et al., 2021; Peralta et al., 2019; Silva, 2016;), their findings are more consistent with Castanheira and Monteiro da Silva (2022) and Adair et al. (2021), who document the non-existence of sex differentials in Peru and several other countries.

This study of motivations and barriers to death registration is valuable for many practical reasons. First, it identifies that even in urban areas with relatively high death registration completeness (e.g., above 80%) there can be substantial underlying disparities. Second, it identifies a range of household-level attributes associated with underregistration of deaths. These diagnostics are crucial in guiding efforts by the Government of Senegal and its community partners in closing remaining gaps in death registration completeness. Two limitations of the study are (a) its inability to directly observe correlates or key drivers of late registration or non-registration of deaths and (b) the data are not able to identify how intra-household dynamics and decision-making shape the propensity for a death to be registered.

In summary, the study by Fall et al. (2021) highlights the important diagnostic potential of census data in evaluating major factors affecting death registration completeness as well as the need for further follow-up studies to pilot interventions that incentivize and facilitate complete and timely death registration among households of lower socioeconomic status.

In the sixth paper in this *Genus* thematic series, Gupta and Mani (2022) construct annual life-tables from death registration and sample registration data by sex for the southern state of Kerala from 2006–2017. Their analysis shows that, for Kerala, mortality data from the civil registration system and sample registration system are consistent and the civil registration provides smoother rates, given its more comprehensive approach. They also note that over a recent 12-year period the quality of death registration data have progressively improved—when considering the amount of missing data on age or sex as well as the quality of age reporting.

Gupta and Mani (2022) make the important point that sustained improvements in the civil registration system in India are now making possible the production of high-quality mortality statistics from the official death registration system in some states of India. This point, also made by Rao and Gupta (2020), has at least three important policy implications. First, the authors demonstrate the importance of routine demographic data quality assessment and completeness measurement in identifying the strength and limitations of both the civil registration system and alternative systems such as sample registration. Second, their findings call for further examination of the viability of using the official death registration data in more states of India when producing official vital statistics instead of solely relying on the sample registration data in all states and union territories of India. Third, the authors demonstrate the feasibility of producing a more detailed array of mortality statistics from civil registration data in India by showing that annual life-tables can be constructed from the civil registration data. This opens up opportunities for deeper analyses in some states and union territories of India beyond the existing limited production of crude death rates, infant mortality rates, and under-5 mortality rates. This paper provides a strong example of how official statistical agencies and researchers can better leverage death registration data to inform a more continuous and detailed understanding of population health dynamics.

In the seventh paper of the thematic series, Haider et al. (2021) explore the level and determinants of death registration in a rural area of Bangladesh—a country with historically low birth and death registration completeness (United Nations, 2021). In their study of the levels and determinants of death registration in rural Bangladesh, the authors found only 1 in 6 adult deaths were registered in a recent 3-year period. Using a retrospective survey administered in the Matlab Health and Demographic Surveillance Site, they document large gender inequalities in registered deaths: 1 in 4 deaths of adult men were registered with the civil registry, compared with 1 in 20 deaths registered for adult women. The primary reasons cited by those who had registered deaths of relatives was the need to secure inheritance or accessing social services. The main reasons for non-registration of deaths, on the other hand, were lack of awareness of the importance and procedural processes of death registration and a lack of strong incentives for next of kin to register a death.

The research by Haider et al. (2021) suggests the need for more research and investigation into community outreach and strengthening of behavioral incentives to register deaths in Bangladesh (Castle et al., 2020; United Nations Statistics Division, 2019a). This work builds on a growing body of important research that leverages the information and research infrastructure of health and demographic surveillance areas when studying the determinants, levels, and consequences of vital event underregistration (Arudo et al., 2003; Byass et al., 2002; Clark, 2004; de Savigny et al., 2018; Garenne et al., 2016; Joubert et al., 2014; Kabudula et al., 2014; Prasartkul & Vapattanawong, 2006). As such, it provides rich detail on the individual-level and household-level dynamics that shape the level and determinants of death registration.

In the eighth paper of the series, Castanheira and Monteiro da Silva (2022), in their examination of Peruvian mortality data, found notable inconsistencies between the death registration data maintained by the Ministry of Health (MINSA), the death registration data maintained by the civil registry (RENIEC), and DHS data. Between 2005 and 2011, the difference between deaths registered in the civil registry and in the Ministry of Health ranged from 8.9% to 27.2%. This stems from the multi-layered nature of institutional responsibilities involved in death notification, death registration, and certification of causes of death (Cobos Muñoz et al., 2020; United Nations, 2014). This also implies that there is much need for caution in using death registration data when compiling mortality statistics and a need for additional investigations into the quality and consistency of mortality data in Peru.

Castanheira and Monteiro da Silva (2022) also address knowledge gaps on the trends in sex differentials in death registries from the health information system and the evolution of the sex gap in adult mortality over the last decade in Peru. Their analysis suggests that the current sex differential in adult mortality estimates and life expectancy might be overstated; they attribute this to an over-reliance on indirect estimates that are based on summary indicators computed from household surveys or population censuses—such as under-5 mortality rates—and model life-tables (Moultrie et al., 2013; Palloni, 1981). They find that the sex gap in life expectancy in Peru spans a wide range, varying from 2 to 5 years, depending on the data source used (e.g., ministry of health death registration data, civil registry death registration data, survey data) and the estimation method employed (death distribution method variants, with different age trim choices, sibling-survival estimation, or direct survey estimation, or model-based estimates). A limitation of their study is that their examination of sex differences in death registration completeness across regions of Peru is limited to Peruvian Ministry of Health data. When assessing death registration completeness in Ecuador, Peralta et al. (2019) found that although sex differentials were relatively small at the national level (68% for males versus 65% for females), they were persistent across almost all administrative regions and as high as 16 percentage points in some localities. Nevertheless, the study by Castanheira and Monterio da Silva (2022) highlights the importance of cross-validation of death registration data with other available data sources on mortality and the need for further technical evaluations.

## The use of incomplete and deficient death registration data to track COVID-19 excess mortality

The final two papers in this *Genus* thematic series, Dorrington et al. (2021) and Lima et al. (2021), document technical challenges and practical solutions to analyzing excess mortality during the COVID-19 pandemic in countries where death registration is incomplete—namely Brazil, Chile, Ecuador, Guatemala, Mexico, Peru, and South Africa. Such analyses have informed public understanding of the COVID-19 pandemic and shaped policy responses (Agrawal et al., 2021; Clarke et al., 2021;  Goldstein & Lee, 2020; Helleringer & Queiroz, 2021; Polyakova et al., 2020; Viglione, 2020). Both papers highlight the importance of timely, public dissemination of death registration data to support continuous assessment of the completeness and quality of the registration data and updates on the mortality effects of the SARS-CoV-2 pandemic.

Dorrington et al. (2021), noting the challenges of incomplete and delayed reporting in the national population register (NPR) and death register of South Africa, demonstrate how weekly excess mortality monitoring is possible when the NPR is systematically updated via the CRVS system. They make use of 10 years of recent data on registered deaths, and employ death distribution methods and comparative validation analysis to identify and adjust for underregistration of both adult and child deaths. However, they also note remaining challenges, spanning lack of engagement with real-time excess mortality estimates by national and provincial authorities, disruptions to birth registration during initial periods of the pandemic that may obscure child mortality, further assessment of baseline mortality calibration across provinces, and localized spatial monitoring of mortality.

Lima et al. (2021) evaluate monthly death registration data in six Latin American countries during the COVID-19 pandemic to understand impact on life expectancy at birth. Similarly to Dorrington et al. (2021), they contend with notable variability in underregistration of deaths, delayed registration issues, and variations in the place of occurrence and place of registration. They use P-scores to measure excess mortality in subnational areas of Brazil, Chile, Ecuador, Guatemala, Mexico, and Peru and examine its relationship with life-table entropy—i.e., the association between the relative challenges in life expectancy with changes in age-specific mortality rates. The importance of such analysis has been demonstrated by Andrasfay and Goldman (2021), who showed the disproportionate impact of COVID-19 mortality on reductions in life expectancy among Black and Latino populations in the United States. The analysis by Lima et al. (2021) also highlights the need for civil registration data in Latin America to be organized and disseminated with covariate information by sex, age, geographic region, ethnicity, and Indigenous identity to ensure equitable responses to the COVID-19 pandemic.

## Conclusions and future research directions

This *Genus* thematic series shows that “Who counts?” is a difficult political, economic and technical question that requires an interdisciplinary approach. The series reminds us that civil registration systems are pivotal for societal inclusion throughout the life-course; they are not just pertinent issues in low-resource settings, but also are unresolved among some of the most vulnerable populations in higher income settings—such as foreign labor migrants in Malaysia and the homeless in Milan, Italy. To realize the promise of “leave no one behind”, civil registration system-strengthening needs to be human-centered in both its design and its implementation. Additional efforts are needed to ensure the engagement with and inclusiveness of vulnerable subpopulations.

This series highlights the need for an interdisciplinary approach to assessing and using civil registration systems and their resulting data. Complementary population data sources, such as population and housing censuses, household surveys, sample register systems, and health and demographic surveillance sites have all been shown to be useful in assessing the completeness and quality of vital event registration and legal identity systems. The papers in the series also note the diverse range of actors involved in civil registration, vital statistics, and legal identity—ranging from national, provincial and local authorities in the health, justice, official statistics, social welfare, and home affairs sectors in addition to civil society. Further, understanding the complex nature of challenges facing CRVS systems requires a multiplicity of methods, ranging from formal demographic analysis, statistical methods, qualitative studies, and mixed-method study designs. The use of multiple methods, as shown in this *Genus* thematic series, illuminates barriers at multiple levels, individual, household, community and societal levels.

Marriage and divorce registration have long been neglected areas of research by demographers when it comes to birth and death registration. Engagement with marriage, separation, and divorce registration data often requires partnership with local and provincial governments, ministries of justice, and the court system—entities that are not yet well-established collaborators of demographers, population scientists, and official statisticians. Also, the relationship between (the lack of) proof of legal identity and access to social protection systems has also received relatively little attention in population sciences. This *Genus* thematic series demonstrates how classical demographic methods, used in mortality estimation and analysis, can be adapted to study the frequency, nature and patterns of marital dissolution. Exclusion from civil registration and legal identity systems can place vulnerable subpopulations in heightened situations of vulnerability and shut them out from social protection schemes and full participation in community life. Also, gender relations and the effect of patriarchal societies influences the nature of vital event underregistration. This underscores the importance of a holistic life-course approach to the strengthening of civil registration, vital statistics, and legal identity systems.

The *Genus* thematic series on CRVS systems spans small-area studies, national analyses, and country comparison studies. These analyses, in turn, provide rich insights into the levels, determinants, and consequences of vital event underregistration. However, they also point to a persistent limitation of existing literature on CRVS: the lack of operations research into the scalability of different CRVS system-strengthening approaches and their generalizability across varied settings (Suthar et al., 2019). Current CRVS improvement frameworks do not yet explicitly incorporate evaluation and operational scale-up into their research and practice (Cobos Muñoz et al., 2018; Vital Strategies, 2021). Recent efforts in maternal and child health have identified the four core components of such evaluation scale-up frameworks as (a) a conceptual model for systems improvement; (b) a set of core monitoring and evaluation indicators; (c) guidelines for systematic documentation of systems improvement initiatives, and (d) standards to support systematic evaluation over time and across locations (Bryce et al., 2011). More research is needed that combines operations research, program and impact evaluation methodology, and demographic measurement to identify what works, when it works, how it works, and why it works when it comes to CRVS system-strengthening initiatives.

## Abbreviations

CRVS: Civil registration and vital statistics system
DHS: Demographic and Health Survey
IUSSP: International Union for the Scientific Study of Population
NPR: National Population Register
SARS-CoV-2: Severe acute respiratory syndrome coronavirus 2
SDG: Sustainable Development Goals
